# Face pareidolia in the brain: Impact of gender and orientation

**DOI:** 10.1371/journal.pone.0244516

**Published:** 2020-12-31

**Authors:** Marina A. Pavlova, Valentina Romagnano, Andreas J. Fallgatter, Alexander N. Sokolov

**Affiliations:** 1 Department of Psychiatry and Psychotherapy, Medical School and University Hospital, Eberhard Karls University of Tübingen, Tübingen, Germany; 2 LEAD Graduate School & Research Network, Eberhard Karls University of Tübingen, Tübingen, Germany; 3 German Center for Neurodegenerative Disorders (DZNE), Medical School and University Hospital, Tübingen, Germany; Tilburg University, NETHERLANDS

## Abstract

Research on face sensitivity is of particular relevance during the rapidly evolving Covid-19 pandemic leading to social isolation, but also calling for intact interaction and sharing. Humans possess high sensitivity even to a coarse face scheme, seeing faces in non-face images where real faces do not exist. The advantage of non-face images is that single components do not trigger face processing. Here by implementing a novel set of Face-n-Thing images, we examined (i) how face tuning alters with changing display orientation, and (ii) whether it is affected by observers’ gender. Young females and males were presented with a set of Face-n-Thing images either with canonical upright orientation or inverted 180° in the image plane. Face impression was substantially impeded by display inversion. Furthermore, whereas with upright display orientation, no gender differences were found, with inversion, Face-n-Thing images elicited face impression in females significantly more often. The outcome sheds light on the origins of the face inversion effect in general. Moreover, the findings open a way for examination of face sensitivity and underwriting brain networks in neuropsychiatric conditions related to the current pandemic (such as depression and anxiety), most of which are gender/sex-specific.

## Introduction

Face pareidolia refers to a tendency of seeing faces in patterns of clouds, shadows, landscapes and houses. A kind of predisposition for seeing faces in non-face images or, in other words, to a coarse face schema (such as two eyes above a mouth) emerges early in lifespan [1]: fetuses in the third trimester of pregnancy [2], human infants [3–6], children aged 5–6 years [7], non-human primates such as the Rhesus monkey [8], domestic chicks [9, 10], and even tortoise hatchlings [11] are reported to demonstrate a preference for face-like stimuli over similar scrambled or presented upside-down configurations and images of other objects that do not elicit face impression. Such preference in species with no parental care advocates the existence of a general mechanism to detect animacy in the natural environment [12]. Brain imaging such as functional magnetic resonance imaging, fMRI [13, 14], magnetoencephalography, MEG [15], and electroencephalography, EEG [16–19] reveal that real faces and face-like non-face images activate similar occipito-temporal brain clusters with a hub in the fusiform face area, FFA (for review, see [20, 21]). Face impression in such images usually arises spontaneously without any efforts. The primary advantage of non-face images is that single components do not automatically prompt face processing [20–26]. In other words, face tuning occurs without being explicitly cued by familiar elements such as eyes. For seeing a face, one has to establish spatial connections between non-face components constituting a coarse face scheme. Alternatively, familiar cues facilitate top-down mechanisms downgrading early stages of face processing. In a nutshell, face pareidolia phenomenon is a valuable tool for experimental investigation of face processing and underpinning neural networks. Research on face tuning is of particular relevance during the current rapidly evolving Covid-19 pandemic leading to social distancing, isolation, and anxiety, but also requiring social integrity and sharing.

For a long time, it has been well recognized that display inversion (180° rotation in the image plane) impairs face processing and facial affect recognition [27–35]. Face inversion leads to an increase in response time along with a lessening in accuracy of familiar faces identification or recognition of a face as a face, and usually drops percent correct by about 15–25% [33, 36, see also 37]. The effect of inversion appears to be not so strong with other types of mono-oriented stimuli such as depictions of cars and houses [33, 38, 39]. The face inversion effect is arising in human infants during the first year of life [34]. One of the most impressive examples of influence of inversion on face perception is the famous Margaret Thatcher illusion, named after the late former British Prime Minister Margaret Thatcher, on whose photograph alterations in perception of facial expression in inverted faces (with canonically oriented face elements such as eyes) were initially demonstrated by Peter Thompson [40].

Upright and inverted faces are presumably processed in qualitatively different ways. With upright oriented faces, holistic processing dominates: different face elements are processed in parallel and then shape a perceptual whole, whereas face inversion leads to a less efficient serial analysis of single features [41–45]. Display inversion provides a proper control for face perception and recognition: the amount of intra-stimulus information with upright and inverted orientations is the same, while display inversion leads to a substantial decrease in face recognition. This experimental manipulation is rather extensively used in face perception research, in particular, in infants and patient populations (e.g., preferential looking with simultaneously presented upright and inverted faces is used instead of tools based on verbal or motor responses that are impossible or difficult to collect) and brain imaging (e.g., the passive viewing paradigm is implemented with a set of upright and inverted faces). Although previous (yet rather scarce) work with face-like non-face images also uses inverted displays [18, 46, 47], it has not been directed at studying the impact of inversion on recognition of face-like non-face images *per se*. Instead, most studies take for granted that display inversion generally deteriorates face impression in such images.

Alongside with display orientation, gender of observer represents an essential variable in face research. It is widely believed that gender differences exist in face processing with female superiority in recognition of facial affect, face gender, and familiarity [48–53]. Yet the experimental evidence is contradictory. It is even less clear whether females surpass on non-affective face perception. Females are reported to be more proficient on facial detection tasks and facial identity discrimination: they outperform males in accuracy when asked to determine whether spatially scrambled images of upright and inverted faces and trees represent faces [54]. By assessing a newly developed tool for face perception investigation, the Face-n-Food test comprising a set of images (composed of food elements such as fruits and vegetables) and bordering on the Giuseppe Arcimboldo style [20–26], female advantage in face tuning was found in a sample of university students [22]. The female brain is reported to be more responsive to non-face face-like images (such as clocks or backpacks) with a greater activation in such areas of the social brain as the right superior temporal sulcus (STS) and Brodmann area 22 [17].

**Fig 1:**
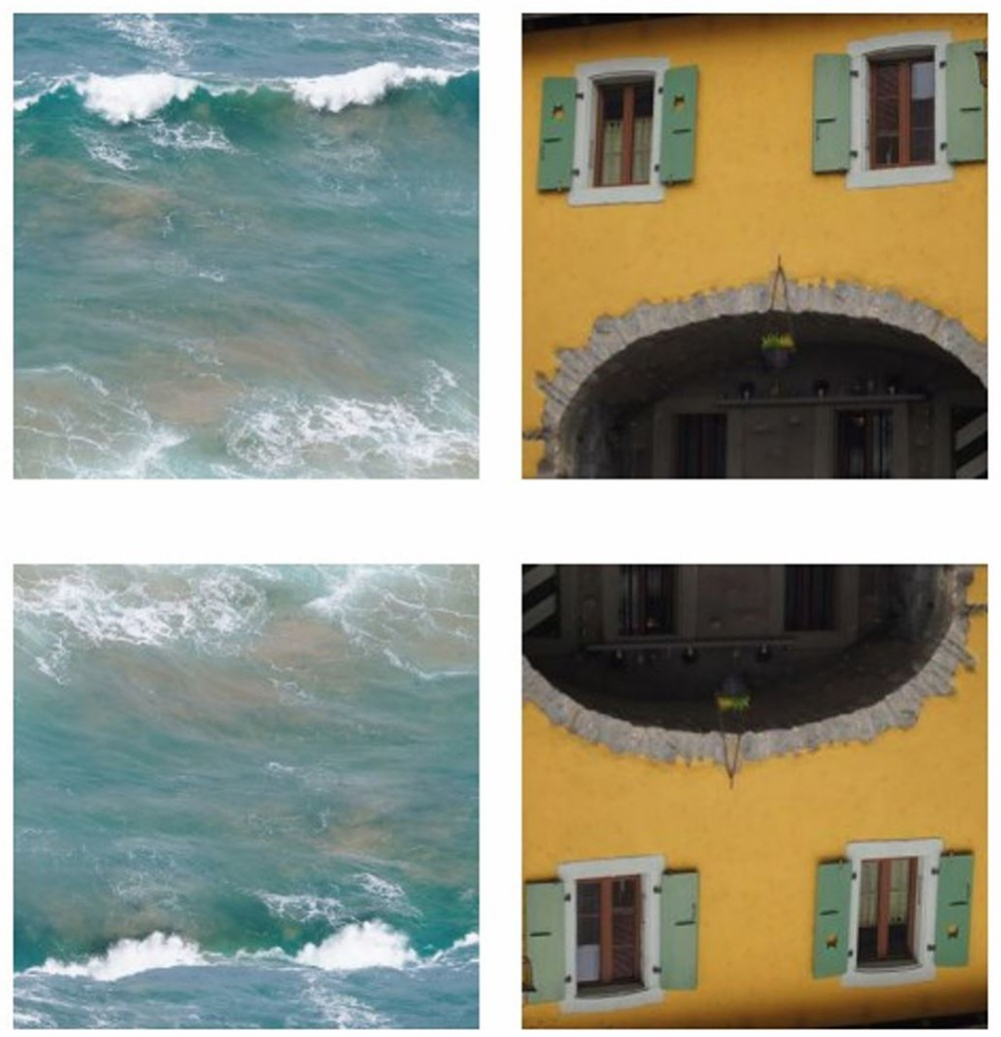
Examples of the Face-n-Thing images with canonical upright (top) and inverted (bottom) display orientation. The image on the left is an example of one of the least resembling a face with upright display orientation, and the image on the right is one of the most resembling a face when presented with upright orientation.

The present exploratory study was designed for investigation of face pareidolia by implementing a novel set of Face-n-Thing images (Fig 1). The aim of this work was twofold: (i) to elucidate how face tuning to non-face images alters with changing display orientation, and (ii) to clarify whether, and if so, how face pareidolia is affected by observers’ gender. To this end, young adult females and males were administered a computer based task with a randomized set of Face-n-Thing images resembling a face (such as sea waves, postboxes, and houses) either with usual upright orientation or inverted 180° in the image plane. We expected the outcome will be beneficial for using display inversion in imaging of the social brain in neuropsychiatric diseases (such as autism, schizophrenia, and depression), most of which are gender-specific [55, 56].

## Methods

### Participants

Forty-nine young adults (25 females and 24 males; aged 18–39 years; students of the University of Tübingen, Germany) were enrolled in the study. None had a history of neurological or psychiatric disorders including autistic spectrum disorders (ASD), schizophrenia, or depression (that potentially may affect visual social cognition) and regular intake of medication. All participants were German natives. We used this inclusion criterion, since some cultural differences were previously reported on a similar Face-n-Food task with non-face images consisting of food elements [26]. Three females turned out to be outliers: even with high individual variability in performance (see Results section), in these participants, face recognition accuracy (face response rate) differed from average for more than 2 standard deviations (SD) and was close to zero. Their data, therefore, were excluded from further data processing. This left 22 female participants aged 24.27±2.60 years (mean±SD) for data processing. Males were aged 24.46±4.99 years (with no age difference between females and males; t(44) = 1.159, p = 0.874, two-tailed, n.s.). Participants were run individually. None had previous experience with such stimuli and tasks. All observers had normal or corrected-to-normal vision. The study was conducted in line with the Declaration of Helsinki and was approved by the local Ethics Committee at the Medical School, Eberhard Karls University of Tübingen. Informed written consent was obtained from all participants. Participation was voluntary and the data were processed anonymously.

### Task and procedure

Participants were presented with a set of Face-n-Thing images (such as houses, clouds, stones, waves, etc. (Fig 1), photographs taken by MAP) eliciting a face impression. Images serving as Face-n-Thing stimuli for the present study had been chosen from these photographs by several experts in face processing. Participants were administered a computer version of the task by using Presentation software (Version 20.3, Neurobehavioral Systems, Inc., Albany, CA, USA). The stimuli subtended a visual angle of 9.8° × 9.8° at an observation distance of 70 cm. The images were pseudo-randomly presented one by one for 1 s with either canonical or inverted orientation in 3 runs with a short break between the runs. In total, each experimental session consisted of 168 trials (14 images × 2 types [original/mirror image] × 2 display orientations [upright/inverted] × 3 runs). In addition, no more than three images with the same orientation (either upright or inverted) appeared consecutively; in this way we prevented a possible adaptation of the visual system to display orientation. Participants were asked to respond upon stimulus offset only. During an inter-stimulus interval (after stimulus offset and till onset of the next stimulus right after participant’s response), a white fixation cross was displayed in the center of the screen for duration jittered from 4 to 6 s. If a participant failed to respond within this period, the next trial automatically started. On each trial, participants had to indicate whether they had an impression of a face or not. They were explicitly told that there were no correct or incorrect responses on the task and they have to rely solely upon their own visual impression. They were asked to respond as fast as possible after the stimulus offset by pressing a respective key (face impression, no face impression). No immediate feedback was provided. With each participant, the testing procedure lasted for about 25–30 min.

### Data processing and analysis

Prior to statistical data processing, all data sets were routinely analyzed for normality of distribution by using Shapiro-Wilk tests with subsequent use either parametric (for normally distributed data) or non-parametric statistics. For not normally distributed data sets, additionally to means and SDs, medians (Mdns) and 95% confidence intervals (CIs) are reported throughout the paper. Inferential statistics was performed by mixed model analyses of variance, ANOVAs, and post-hoc pairwise comparisons by using Tukey’s honestly significant difference (HSD) tests with software package JMP (version 14, SAS Institute Inc. 2019, Cary, NC).

## Results

**Fig 2:**
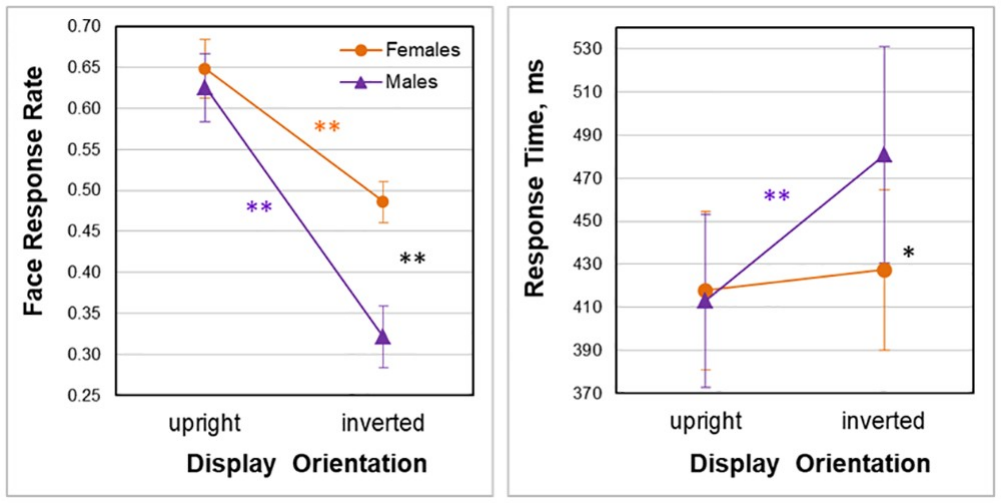
Effect of inversion on the face tuning. Mean response rate (left) and mean response time (right) of the face responses to the Face-n-Thing images with canonical (upright) and inverted display orientations in females (orange blobs) and males (violet triangles). Vertical bars represent ±SEM. Double asterisks indicate significant differences: black, gender differences, orange, display orientation effect in females, and violet, display orientation effect in males (p < 0.05). Single asterisk indicates a tendency of females to provide face response faster than males with display inversion (p = 0.079).

Individual data on face recognition accuracy (face response rate) were submitted to a two-way mixed model ANOVA with a within-subject factor Display Orientation (upright/inverted) and between-subject factor Gender of Observer (female/male). Both the main effects of Display Orientation (F(1;180) = 57.598, p < 0.001; effect size, eta squared η^2^ = 0.567; with greater face response rates for upright orientation) and Gender of Observer (F(1;180) = 9.498, p = 0.004; effect size, η^2^ = 0.178; with greater face response rates in females) were highly significant with a significant interaction between these factors (F(1;180) = 5.405, p = 0.025; effect size, η^2^ = 0.110). Post-hoc analyses indicated that: (i) whereas with upright display orientation, there were no gender differences in face response rate (for females, 0.65±0.17, mean±SD; for males, 0.63±0.20; t(44) = 0.54, p = 0.95, two-tailed HSD test throughout, n.s.), with display inversion, face impression from face-like non-face images was substantially impeded in males as compared to females (for females, 0.49±0.12, for males, 0.32±0.19; t(44) = 3.82, p = 0.002, effect size, Cohen’s d = 1.06; two-tailed HSD test, here and further corrected for multiple comparisons). [Notably, the effect of gender on face resemblance of inverted Face-n-Thing images appeared to be more pronounced with images more resembling faces with upright orientation (for a half of all images less resembling face when presented upright, U = 170.5, p < 0.026; for males, 0.13±0.12 (Mdn, 0.11; 95% CI, 0.08 to 0.18); for females, 0.29±0.16; for a half of all images more resembling face when presented upright; U = 2, p < 0.0001; for males, 0.52±0.11; for females, 0.75±0.12). In addition, in females, a strong positive correlation was found between face response rate with upright and inverted display orientations r(22) = 0.620]; and (ii) display inversion deteriorated face recognition in both females (t(21) = 3.64, p = 0.004, effect size, d = 1.087) and males (t(23) = 7.17, p < 0.001, effect size, d = 1.589). In females, display inversion resulted in a drop of face impression from the Face-n-Thing images by 25%, whereas in males face impression fell down by 48.64% (Fig 2).

Individual data on response time (for trials, on which images elicited face impression) were also submitted to a two-way mixed model ANOVA with a within-subject factor Display Orientation (upright/inverted) and between-subject factor Gender of Observer (female/male). The main effects of Display Orientation (F(1;180) = 6.369, p < 0.015, effect size, η^2^ = 0.127) was significant (with longer response times for inverted orientation), whereas neither main effect of Gender of Observer (F(1;180) = 2.526, p = 0.119, n.s.), nor interaction between these factors (F(1;180) = 3.620, p = 0.064, n.s.) were observed. Post-hoc analyses showed that: (i) with upright display orientation, there was no gender difference in response time (for females, 417.81±173.20 ms; for males, 413.02±197.40 ms; t(44) = 0.22, p = 0.996, n.s.), while with display inversion, there was a tendency for females to respond faster than males when they had a face impression (for females, 427.32±174.96 ms; for males, 480.78±246.07 ms; t(44) = 2.47, p = 0.079); and (ii) in females, difference in face response time to upright and inverted images was non-significant (t(21) = 0.43, p = 0.973, n.s.), whereas males were slower in response to images presented upside-down (t(23) = 3.20, p = 0.013, effect size, d = 0.304). In females, display inversion yielded a drop in response time by 2.28%, whereas in males by 16.41%.

In both females and males, images that more often elicited face impressions when presented with canonical upright orientation were also more recognizable as faces with display inversion: Pearson product moment correlation, r(22) = 0.937, p < 0.001, for males; Spearman’s rho ρ(20) = 0.927, p < 0.001, for females.

## Discussion

In the present study, we explored a potential impact of display orientation and gender of observer on face pareidolia, our ability to seeing faces in face-like non-face images such as clouds or toasts. With this purpose in mind, healthy females and males were administered a computer task with a pseudo-randomized set of the Face-n-Thing images (photographs of waves, houses, clouds, etc.) in different degree resembling a face. All of them contain a coarse face schema such as two elements (eyes) above an element on a mouth place. As compared to depictions of real faces consisting of familiar elements (e.g., a nose), the benefit of these images is that single elements (such as windows) do not explicitly trigger face processing. The outcome indicates: (i) In both female and male observers, face impression is substantially impeded by display inversion in terms of accuracy and response time; (ii) With display inversion, the Face-n-Thing images evoke face impression in females significantly more often than in males, whereas with upright orientation, there are no gender differences in accuracy and response time. Display inversion cuts face impression by 25% in females, and almost by 50% in males. Furthermore, the effect of gender on face resemblance of inverted Face-n-Thing images appears to be more pronounced with images more resembling faces with upright orientation; and (iii) In both females and males, the Face-n-Thing images that are less recognizable as faces with upright orientation, produce also fewer face responses with display inversion.

### Display inversion effect in Face-n-Thing images

One of the most influential accounts for the face inversion effect is the holistic processing hypothesis [57–60]. According to this assumption, display inversion detrimentally affects a holistic representation of a face as a whole (*Gestalt*) thereby forcing less efficient strategies by serial, rather than parallel, processing of local features and elements [41–45, 58, 61]. Face inversion effect is also frequently explained by the lack of visual expertise: in daily life, people use to see faces with upright orientation, with the same configurational pattern (eyes, a nose, and a mouth from up to down), and consequently they develop a predisposition for this canonical orientation [29, 31, 33]. This explanation is closely related to the face-scheme incompatibility model [62] that mainly refers to early stages of face processing: display inversion simply disrupts the rough face representation in the brain such as *two eyes are above a mouth*.

Upright and inverted faces apparently follow dissimilar ways of brain processing. Brain imaging indicates that upright faces engage face-specific neural networks comprising occipito-temporal cortices (with a hub in the FFA) and evoke specific brain activation at 170 ms after stimulus onset primarily in the right brain hemisphere [45, 63], while a greater number of brain regions (which are also engaged in processing of non-face images) is recruited for processing of inverted faces [64]. Obviously, the networks for processing of upright and inverted faces are topographically overlapping: for example, the occipital face area (OFA) is involved in the processing of both upright and inverted faces [65, 66]. MEG and EEG underscore the main difference in processing of upright and inverted faces in the time course of unfolding brain activation rather than in topography of engaged brain regions solely [18, 29]. Latencies of brain responses to inverted compared to upright faces are longer in the right and shorter in the left hemisphere [67].

By using novel non-face face-like Face-n-Thing images, the present work yields a further experimental support for the face inversion effect: for both female and male observers, display inversion hampers face resemblance of images as well as processing speed. Bearing in mind that the Face-n-Thing images contain only a rough face schema without any single element signaling face occurrence, it appears that our findings most likely speak for the face-scheme incompatibility model [62]. Indeed, in the absence of clear face-impression triggering cues, with display inversion a face scheme does not work properly and, therefore, some efforts (such as image normalization) are required for acquisition of a face impression. In accord with this, the outcome of the present study indicates that in both females and males the Face-n-Thing images that are less recognizable as faces with upright orientation, elicit also fewer face responses with display inversion.

Previous work with non-face face-like stimuli (similar to the Face-n-Thing images used in the present study [17]), prototype faces containing just a few blobs in accordance with a simplified coarse face scheme [68], and with Arcimboldo-like images [46, 47] was not directed at studying the impact of display inversion on face processing *per se*. Human fetuses in the third trimester of pregnancy are reported to be responsive to a coarse face schema, since they are ‘engaged’ merely with face prototypes presented with upright orientation rather than with the same stimuli inverted 180° [2]. By using display inversion, human infants [5] and children aged 5–6 years [7] were demonstrated to exhibit visual orienting towards upright face-prototype stimuli. Healthy children aged 24–60 months more often direct their first fixation towards face-like upright as compared with inverted stimuli than their peers with autism [69]. By 7–8-month-old infants, the original Arcimboldo portraits are visually preferred over the same inverted paintings [46].

Brain imaging uses display orientation primarily for contrasting brain activation in response to upright face-like images as compared to inverted stimuli. For instance, compared with the same upside-down paintings, original Arcimboldo portraits yield fMRI brain activation in the right FFA and posterior superior temporal area [70]. The Arcimboldo portraits elicit larger N170 amplitude of the event-related potential, ERP, than inverted paintings [71]. Near infra-red spectroscopy (NIRS) conducted in 7-8-month-old infants indicates that in response to upright Arcimboldo portraits compared with images of single vegetables, the concentration of oxy-Hb increases in the left temporal area, while inverted portraits do not produce such effect [46]. With upright orientation, N170 amplitudesdiffer between Arcimboldo portraits and natural faces in the left (but not right) occipito-temporal region, whereas with inversion, in both hemispheres, N170 amplitudes do not differentiate Arcimboldo paintings from objects, but are reduced compared to faces [47]. This finding dovetails well with common visual experience with Arcimboldo portraits: visual processing is affected by display inversion in such a way that they are perceived simply as a composition of fruits and vegetables. Display inversion decreases face-likeness scores of faces, Arcimboldo portraits, and face-like images of cars and insects [18]. EEG shows that while with canonical display orientation, no difference occurs in the amplitude of P1, N170, and N250 components of ERPs for all types of stimuli, display inversion leads to a significant increase in the N170 amplitude in the right hemisphere for real faces solely. Furthermore, the N170 amplitude differentiates between inverted images of face-like insects and Arcimboldo portraits: it is greater for face-like images of insects [18].

### Gender matters for the inversion effect

The eye-catching finding of the present study is that the face inversion effect in non-face face-like images is profoundly modulated by observer’s gender: females more often and faster reported face resemblance in response to the Face-n-Thing images presented upside-down. To our knowledge, this is the first report about gender differences in the face inversion effect with face-like non-face images. To our surprise, however, we found only a very few studies on gender impact on the face inversion effect even with depictions of real faces. For example, in a large sample of young Italian adults, women are reported to perform significantly better than men on both the upright and inverted versions [72] of the Cambridge Face Memory Test [37] that requires matching unfamiliar faces and recognizing previously seen faces. This is even more arresting if one takes into account that investigation of this issue would shed light on the origins of the face inversion effect. Indeed, if with upright faces and face-like images, (i) females are generally reported to possess predominantly holistic style of face processing [22, 23, 73, 74, but see 75], and (ii) display inversion ultimately impedes holistic face processing (see *Section Display inversion effect in Face-n-Thing images* above), then the face inversion effect should be more evident in females. Yet it was not the case in the present study with non-face images as well as in the study by Albonico and colleagues with real faces [72]. Clarification of this issue calls for further experimental work.

### Lack of gender differences with upright orientation

In the present study, no gender differences were observed in processing (both accuracy and speed of face responses) of the Face-n-Thing images presented with canonical (upright) display orientation. Only a very few earlier studies were directed at investigation of this issue. By using upright face-like stimuli similar to the Face-n-Thing images in the present study, the female social brain was shown to be more face responsive, with greater activation in several areas such as the right STS and Brodmann area 22, though sex differences were absent at earlier stages of face processing [17]. Gender differences at behavioral level were not reported, since the task (participants were asked to respond to images of animals presented along with faces and face-like non-face images) did not allow for such analysis.

Earlier work of our lab points to gender differences in spontaneous recognition of the Face-n-Food Arcimboldo-like images [22]. Face resemblance is closely connected to gender-specific impression: images most resembling a face elicit more female-face responses in both female and male observers. In females only, face resemblance is positively linked to face likability [23]. Yet gender effects can be modulated by culture: no gender differences in the face tuning to the Face-n-Food images were found in young adults of the French-speaking part of Switzerland. Swiss males demonstrate higher face sensitivity than their German peers, while young Swiss and German females do not display any differences in face tuning [26]. (In general, culture can profoundly affect face processing [76, 77]). The lack of gender differences in face tuning to the Face-n-Thing as compared to the Face-n-Food images may be explained by diverse mechanisms underlying face resemblance of these types of stimuli. The other possible account may be of methodological origin: here each Face-n-Thing image was presented to observers several times in a pseudo-randomized order, whereas in the Face-n-Food paradigm, the images were presented in the fixed, predetermined order from the least to most evoking a face impression. The latter experimental procedure may be more sensitive to uncovering gender differences (as well as group differences in general). Alternatively, a coarse face scheme may be, at least, partly, sex-independently hardwired in the brain.

### Implications for future research

Along with body language reading, proficient face perception represents an essential component of social competence [1, 55, 56, 78–84]. This ability is reported to be aberrant in most neuropsychiatric conditions such as autism and schizophrenia [55, 56, 79, 85]. Since face tuning in face-like non-face images occurs without being explicitly fostered by familiar elements, these stimuli represent a valuable tool for investigation of face processing [20–26]. The usage of unfamiliar images entails benefits for studying clinical populations [76].

In last years, face-like non-face (Face-n-Food) images were efficiently used in our lab for investigation of the face sensitivity in a number of neurodevelopmental and psychiatric conditions such as autism spectrum disorders [25], Down syndrome [20], Williams syndrome [23], schizophrenia [21], and depression [86]. This work revealed substantial (though rather specific for every single disease) deficits in the face tuning in most of these patient populations (for comparative analysis, see [21]). Individuals with premanifest Huntington’s disease (characterized by aberrant social cognition [87]) show a decrease in the N170 component of ERP elicited by the face-like non-face images, and this decline is positively linked with the number of recognition errors, severity of apathy and global cognitive abilities [88]. In Parkinson’s patients (mostly males), the latencies and amplitudes of N170 and vertex positive potential (VPP) ERP responses to both faces and face-like stimuli are increased, and the amplitude of N250 responses is decreased as compared to healthy controls [89]. In patients with migraine, alterations in N170 and VVP are reported during perception of non-face face-like stimuli [90].

To summarize this section, the outcome of the present study helps to clarify the following issues: (a) Display inversion of face-like non-face images may be a valuable experimental manipulation providing for a proper control for face perception and brain processing: inverted images more often elicit non-face impression than upright displays, whereas they consist of the same number of elements representing the same relative spatial arrangement; and (b) possible gender differences in visual perception of inverted face-like non-face images should be taken into account when conceiving and designing studies and elaborating data processing: whereas in males, display inversion efficiently prevents face impression, in females, this effect is much less pronounced. This also may lead to an insufficient number of trials entering (in particular, MEG) data processing in females as compared to males.
